# Hormonal Predictors of Abnormal Luteal Phases in Normally Cycling Women

**DOI:** 10.3389/fpubh.2018.00144

**Published:** 2018-05-24

**Authors:** Saman H. Abdulla, Thomas P. Bouchard, Rene A. Leiva, Phil Boyle, Jean Iwaz, René Ecochard

**Affiliations:** ^1^Hospices Civils de Lyon, Service de Biostatistique-Bioinformatique, Lyon, France; ^2^Université de Lyon, Lyon, France; ^3^Université Lyon 1, Villeurbanne, France; ^4^Centre National de la Recherche Scientifique, UMR 5558, Laboratoire de Biométrie et Biologie Évolutive, Équipe Biostatistique-Santé, Villeurbanne, France; ^5^Department of Family Medicine, University of Calgary, Calgary, AB, Canada; ^6^CT Lamont Primary Health Care Research Centre, Bruyère Research Institute, Ottawa, ON, Canada; ^7^Department of Family Medicine, University of Ottawa, Ottawa, ON, Canada; ^8^International Institute for Restorative Reproductive Medicine, Dublin, Ireland

**Keywords:** menstrual cycle, luteal phase, ultrasound, predictor, estrone-3 glucuronide, pregnanediol-3- alpha-glucuronide, luteinizing hormone, follicle stimulating hormone

## Abstract

**Objective:** Explore potential relationships between preovulatory, periovulatory, and luteal-phase characteristics in normally cycling women.

**Design:** Observational study.

**Setting:** Eight European natural family planning clinics.

**Patient(s):** Ninety-nine women contributing 266 menstrual cycles.

**Intervention(s):** The participants collected first morning urine samples that were analyzed for estrone-3 glucuronide (E1G), pregnanediol-3- alpha-glucuronide (PDG), follicle stimulating hormone (FSH), and luteinizing hormone (LH). The participants underwent serial ovarian ultrasound examinations.

**Main Outcome Measure(s):** Four outcome measures were analyzed: short luteal phase, low mid-luteal phase PDG level (mPDG), normal then low luteal PDG level, low then normal luteal PDG level.

**Results:** A long preovulatory phase was a predictor of short luteal phase, with or without adjustment for other variables. A high periovulatory PDG level was a predictor for short luteal phase as well as normal then low luteal PDG level. A low periovulatory PDG level predicted low mPDG and low then normal luteal PDG level, with or without adjustment for other variables. A small maximum follicle predicted normal then low luteal PDG level, with or without adjustment for other variables. The relationship between small maximum follicle size and short luteal phase or small maximum follicle size and low mPDG was no longer present when the regression was adjusted for certain characteristics. A younger age at menarche and a high body mass index were both predictors of low mPDG.

**Conclusion:** Luteal phase abnormalities exist over a spectrum where some ovulation disorders may exist as deviations from the normal ovulatory process.This study confirms the negative impact of a small follicle size on the quality of the luteal phase. The occurrence of normal then low luteal PDG level is confirmed as a potential sign of luteal phase abnormality.

## Introduction

Several authors have reported a variety of hormonal profiles in normally fertile women. Moreover, a continuum from normal to abnormal cycles exists (1–6). Some ovulation disorders may represent minor deviations from the regular ovulatory process. We evaluated whether factors associated with luteal phase deficiency are also predictors of various minor luteal phase deficiencies in normally cycling women.

Several luteal phase characteristics have been considered as signs of luteal phase deficiency (7). The more frequently cited are the length of the luteal phase [type 1 according to Hilgers (8)] and the average level of progesterone (Hilgers' type 2) [see also (3, 9)]. Others are related to the duration of the plateau of progesterone level: an early drop (Hilgers' type 3) or a delayed increase (Hilgers' type 4) is a sign of abnormal luteal phase. Hilgers has also proposed a low level of estrogens during the luteal phase (type 5).

If all of Hilgers' signs were applied to normal cycles, few would be classified as abnormal. If these signs were evaluated individually, they may be assessed along with other thresholds to identify the spectrum of suboptimal luteal phases.

In the mid-nineties, a large observational study of normally fertile women collected a high number of daily urine hormone measurements together with ultrasound-confirmed ovulation days. Due to confidentiality agreements, the data regarding the luteal phase could not be disclosed at that time but only recently (6). In the present study, a new analysis of this dataset was performed to assess the place of relationships between the characteristics of the participants, the menstrual cycles, and the periovulatory phase in assessing potential signs of suboptimal luteal phase. A multivariate analysis was carried out to determine probable direct and indirect links; i.e., classify the predictors as independent or not independent.

## Materials and methods

### Ethics approval statement

The protocol was approved by the Comité Consultatif de Protection des Personnes dans la Recherche Biomédicale (Lyon, France). All subjects gave written informed consent in accordance with the Declaration of Helsinki.

### Subjects

Women were recruited between 1996 and 1997 from eight natural family planning clinics located in France, Italy, Germany, Belgium, and Spain and the data were collected during these 2 years. The inclusion criteria were: age 19–45 years and previous menstrual cycle lengths of 24–34 days. The exclusion criteria were: a consistent history of anovulatory cycles, infertility, or active hormonal treatment for infertility in the past 3 months, use of hormonal contraception or hormone therapy in the past 3 months, abnormal cycles (polycystic ovarian syndrome or luteal phase deficiency), hysterectomy, tubal ligation(s), or pelvic inflammatory disease. In addition, the study excluded runners and breastfeeding or postpartum mothers (<3 months). The dataset included thus 107 women who contributed 326 cycles (i.e., three cycles per woman, on average).

We restricted the present study to those cycles with ultrasound-identified ovulation day; i.e., 283 out of 326 cycles. Seventeen cycles with luteal phase lengths of more than 17 days were arbitrarily considered as possible pregnancies and excluded. This left 266 cycles of 99 women for the present analysis; i.e., 82% of the cycles and 93% of the women.

### Ultrasound investigations

Serial transvaginal ovarian ultrasounds with follicle measurement were performed by a single physician per center. Ovarian scanning started on the first day women observed cervical mucus or when an LH surge was detected by an LH home test (Quidel Corporation, San Diego, CA, USA.), whichever occurs first. Scanning was performed every other day until a follicle reached 16 mm and then daily until evidence of ovulation; this determined the ultrasound day of ovulation, US-DO. The largest follicle observed by ultrasonography during the preovulatory—follicular—phase was identified as “small” when its largest diameter was <20 mm.

### Hormonal investigations

Daily-collected early morning urine samples were assayed for quantitative detection of follicle-stimulating hormone (FSH), luteinizing hormone (LH), estrone-3-glucuronide (E1G), pregnanediol-3α-glucuronide (PDG) using time-resolved fluorometric immunosorbent assays (WallacDelfia®, PerkinElmer, Norwalk, CT, USA). E1G and PDG are, respectively, the urine metabolites of estrogens and of progesterone. Hormone levels were estimated at three periods of the menstrual cycle: at day 3 ± 1 of the cycle for the early follicular phase, at US-DO ± 1 for the periovulatory phase, and at mid-luteal phase as average level over US-DO + 5, US-DO + 7, and US-DO + 9 (called mPDG). All the assays were run in duplicates, averaged, and adjusted for urine creatinine.

### Proposed predictors

The study considered three sets of predictors: (1) five general characteristics: age, age at menarche, body mass index, sports activity, and tobacco smoking; (2) five preovulatory characteristics: preovulatory phase length (from beginning of menses to the US-DO, included) and early follicular phase E1G, PDG, LH, and FSH levels as described above; and (3) five periovulatory characteristics: small maximum follicle size and periovulatory phase E1G, PDG, LH, and FSH levels.

### Outcome measures

We created four binary variables to qualify the luteal phase. These four outcome measures were used to identify potential signs of suboptimal luteal phase. These outcomes measures stem from the five types of luteal deficiency defined by Hilgers (8) but were extended to include borderline deficiencies that may exist in normally fertile women. These four outcome measures are defined as follows:

- Outcome 1: Short luteal phase; i.e., <12 days from US-DO + 1 to the day before menses (for Hilgers, this delay is ≤8 days).
- Outcome 2: mPDG <10 μg/mg Cr. This range was arbitrarily chosen to select nearly one third of the menstrual cycles (those with the lowest PDG levels), thus maintaining a sufficient statistical power.
- Outcome 3: Normal then low luteal PDG level; i.e., US-DO + 9 level <10 μg/mg Cr then average level over US-DO + 5 and US-DO + 7 ≥10 μg/mg Cr (in Hilgers' work, this corresponds to “≥50 percent drop on peak day + 9 and peak day + 11”).
- Outcome 4: Low then normal luteal PDG level; i.e., level at US-DO + 5 <10 μg/mg Cr then average level over US-DO + 7 and US-DO + 9 ≥10 μg/mg Cr.

### Statistical analyses

A descriptive analysis expressed the binary variables as percentages and showed histograms that represent the distributions of the luteal phase lengths and the mPDG levels.

Each of the four outcome measures was considered as a binary variable. Some of the predictors were also binary (sports activity, tobacco smoking, and small maximum follicle size), whereas others were continuous quantitative variables. Some of the latter quantitative predictors were asymmetrically distributed (E1G, PDG, LH, and FSH levels); their values were log-transformed before analysis.

Then, logistic regressions were used to assess the prediction of each outcome measure by each predictor; i.e., in each logistic regression, the dependent variable was one of the outcome measures and the independent variable one of the predictors. When the predictor was binary, these regressions were similar to two-by-two tables: an odds ratio was then used to quantify the intensity of the relationship between the predictor and the outcome. The 95% confidence interval of the odds ratio was used to assess its statistical significance. To confirm the statistical significance, we also provide the likelihood ratio test and the corresponding *p*-value. For homogeneity, we used also odds ratios to express the prediction of the binary outcomes by the quantitative predictors. In this case, the odds ratio expresses the increase of the frequency of the outcome for each increase of one unit of the predictor (e.g., a 1 kg/m2 increase of the body mass index). These univariate analyses were used to assess the relationships between the predictors and the outcomes before adjustment for potential confounding effects.

Then, a multivariate logistic regression was used to adjust for potential confounding effects and check whether the predictors were independent predictors or not. At this step, the dependent variable was one of the outcome measures and the independent variables were the studied predictor and other predictors suspected of being potential confounders. To be systematic, we used successively the three groups of predictors as potential confounders. When the relationship between a predictor and an outcome remained significant after adjustment, the predictor was considered to be an independent predictor.

All statistical analyses were performed with SPSS software version 21.0. A *P* < 0.05 was considered for statistical significance.

### Availability of materials

The raw data supporting the conclusions of this manuscript will be made available by the authors, without undue reservation, to any qualified researcher.

## Results

### Demographics

Table 1 presents some characteristics of the women. In this population, the average BMI was low as well as the proportion of regular smokers. Only 7 out of 99 were older than 40 years and 19 were nulliparous.

**Table 1:** Selected characteristics of the participants.

**Characteristic**	**Mean (*SD*) or %**	**Minimum**	**Maximum**
Age (years)	32.66 (5.87)	19	44
Body mass index (kg/m2)	21.26 (2.61)	17.12	28.34
Age at menarche (years)	13.24 (1.65)	9	17
Sports activity	56.8		
Regular tobacco smoking	10.5		

### Descriptive analysis

**Figure 1:**
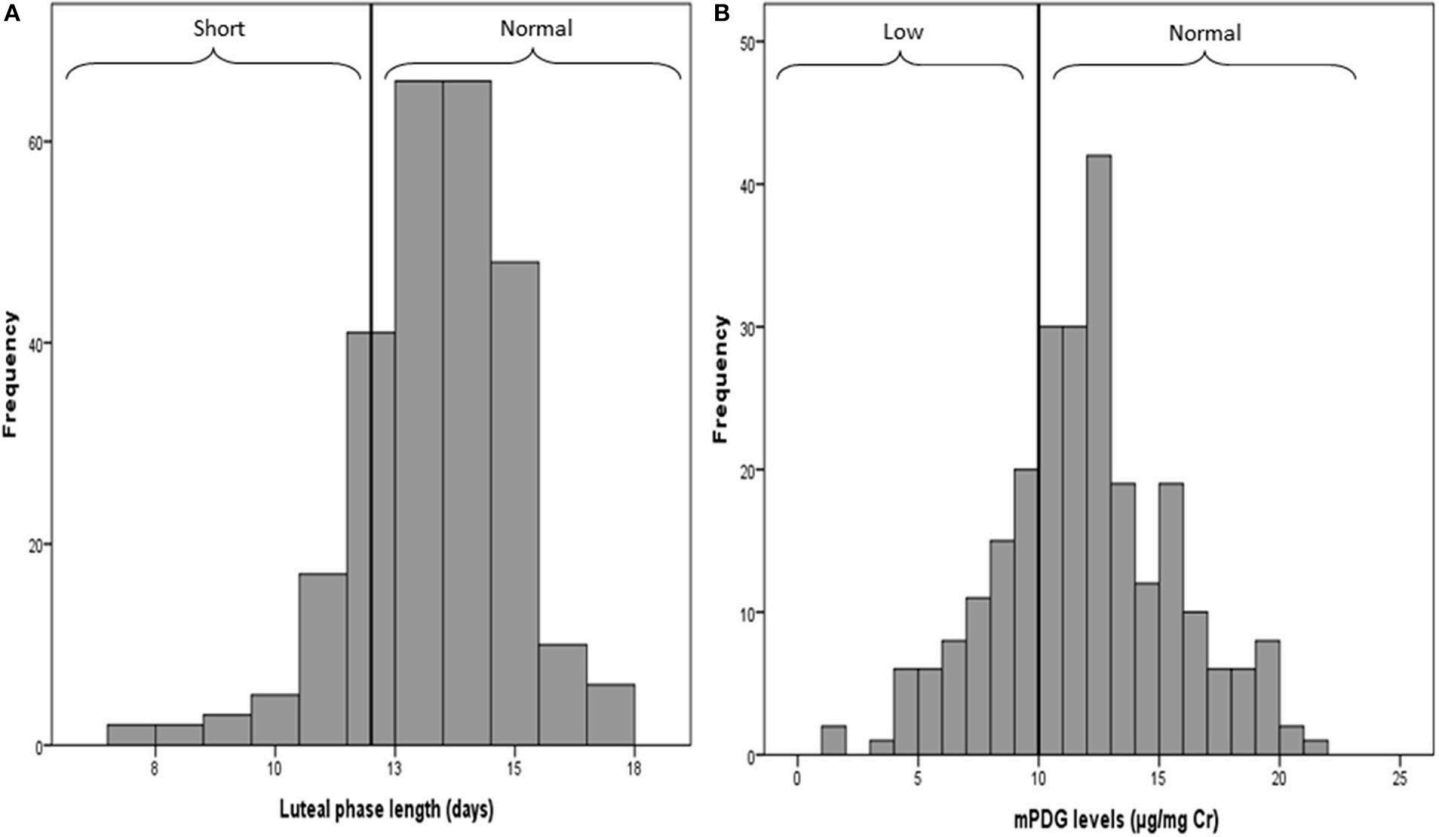
Distributions of the luteal phrase lengths **(A)** and the mean PDG levels **(B)**. Values 12 and 10 are the thresholds used to define short vs. long luteal phase and low vs. high PDG level.

Figure 1A shows an asymmetric distribution (skewed to the left) due to nearly 11% of cycles with short luteal phases (<12 days). Figure 1B shows a symmetric distribution of mPDG levels where 69 cycles (26%) had a mPDG level <10 μg/mg Cr, 34 cycles (12%) had normal then low PDG levels, and 54 cycles (21%) had low then normal PDG levels.

The relationship between early follicular PDG level and, respectively, periovulatory PDG and mPDG, were 0.61 and 0.37. The relationship between periovulatory PDG level and mPDG was 0.34 and that between BMI and age at menarche was −0.17. All of these relationships were statistically significant.

### Results of the univariate analyses

Before adjustment, several predictors were found significant in each of the four outcomes measures (See Table 2 for Outcomes 1 and 2 and Table 3 for Outcomes 3 and 4).

**Table 2:** Multivariate regression analysis of general characteristics as well as preovulatory and periovulatory characteristics on Outcomes 1 and 2 of the luteal phase characteristics.

**Characteristics**	**Short luteal phase**				**Low mPDG**			
	**Without adjustment**	**Adjusted for general characteristics**	**Adjusted for preovulatory characteristics**	**Adjusted for periovulatory characteristics**	**Without adjustment**	**Adjusted for general characteristics**	**Adjusted for preovulatory characteristics**	**Adjusted for periovulatory characteristics**
**GENERAL**^a^								
Age		0.92 (0.86, 1.00)						
		[4.31; 0.04]^b^						
Age at menarche					0.81 (0.68, 0.97)		0.81 (0.68, 0.97)	0.81 (0.68, 0.97)
					[5.79; 0.02]		[5.78; 0.01]	[5.57; 0.02]
Body mass index					1.15 (1.03, 1.28)		1.15 (1.03, 1.29)	1.14 (1.02, 1.28)
Body mass index					[6.39; 0.01]		[6.08; 0.01]	[5.68; 0.02]
**PREOVULATORY**^c^								
Preovulatory phase length	1.45 (1.23, 1.71)	1.47 (1.23, 1.77)	1.45 (1.23, 1.71)	1.44 (1.23, 1.71)				
	[25.66; 0.000]	[24.3; 0.000]	[25.66; 0.000]	[25.62; 0.000]				
Early follicular phase PDG					0.29 (0.16, 0.55)	0.39 (0.21, 0.72)	0.29 (0.16, 0.55)	
					[17.4; 0.000]	[10.04; 0.002]	[17.4; 0.000]	
**PERIOVULATORY**^d^								
Small max. follicle size	2.92 (1.33, 6.40)		2.61 (1.13, 6.03)		1.84 (1.02, 3.33)			2.49 (1.32, 4.73)
	[6.85; 0.009]		[4.91; 0.03]		[3.99; 0.05]			[7.8; 0.005]
Periovulatory phase PDG	3.71 (1.66, 8.27)	3.41 (1.46, 7.94)	2.33 (1.02, 5.31)	3.82 (1.69, 8.57)	0.30 (0.16, 0.54)	0.36 (0.19, 0.66)	0.3 (0.16, 0.54)	0.29 (0.16, 0.55)
	[10.98; 0.001]	[8.83; 0.003]	[4.28; 0.04]	[11.32; 0.001]	[17.9; 0.000]	[11.99; 0.001]	[17.91; 0.000]	[17.53; 0.000]

a *The five general characteristics tested were: age, age at menarche, body mass index, sports activity, regular smoking*.

b *The values shown (red text) are the statistically significant ORs (95% confidence intervals) and [LR; p-value]*.

c *The five preovulatory characteristics tested were: preovulatory phase length and early follicular phase E1G, PDG, LH, and FSH levels*.

d *The five periovulatory characteristics tested were: small maximum follicle size and periovulatory phase E1G, PDG, LH, and FSH levels*.

**Table 3:** Multivariate regression analysis of general characteristics as well as preovulatory and periovulatory characteristics on Outcomes 3 and 4 of the luteal phase characteristics.

**Characteristics**	**Normal then low PDG during the luteal phase**				**Low then normal PDG during the luteal phase**			
	**Without adjustment**	**Adjusted for general characteristics**	**Adjusted for preovulatory characteristics**	**Adjusted for periovulatory characteristics**	**Without adjustment**	**Adjusted for general characteristics**	**Adjusted for preovulatory characteristics**	**Adjusted for periovulatory characteristics**
**GENERAL**^a^								
Body mass index								0.85 (0.74, 0.98)
								[5.38; 0.02]
**PREOVULATORY**^b^								
Early follicular phase PDG				0.16 (006, 0.43) [13.64; 0.000]^c^	0.4 (0.22, 0.76) [8.56; 0.003]	0.37 (0.18, 0.74) [8.83; 0.003]	0.4 (0.22, 0.76) [8.56; 0.003]	
				[13.64; 0.000]^c^	[8.56; 0.003]	[8.83; 0.003]	[8.56; 0.003]	
**PERIOVULATORY**^d^								
Small max. follicle size	3.21 (1.50, 6.87)	3.75 (1.63, 8.61)	3.21 (1.50, 6.87)	2.71 (1.24, 5.92)				
Small max. follicle size	[8.82; 0.003]	[9.63; 0.002]	[8.82; 0.003]	[6.11; 0.01]				
Periovulatory phase PDG	3.39 (1.55, 7.4)	2.96 (1.34, 6.55)	3.39 (1.55, 7.4)	3.39 (1.55, 7.4)	0.24 (0.12, 0.48	0.18 (0.08, 0.4)	0.24 (0.12, 0.48)	0.24 (0.12, 0.48)
	[10.08; 0.001]	[7.79; 0.005]	[10.08; 0.001]	[10.08; 0.001]	[19.58; 0.000]	[22.53; 0.000]	[19.58; 0.000]	[19.58; 0.000]

a *The five general characteristics tested were: age, age at menarche, body mass index, sports activity, regular smoking*.

b The five preovulatory characteristics tested were: preovulatory phase length and early follicular phase E1G, PDG, LH, and FSH levels.

c The values shown (red text) are the statistically significant ORs (95% confidence intervals) and [LR; p-value].

d *The five periovulatory characteristics tested were: small maximum follicle size and periovulatory phase E1G, PDG, LH, and FSH levels*.

A long preovulatory phase, a small maximum follicle size, and a high PDG level during the periovulatory phase were all significantly associated with a higher risk of short luteal phase (Outcome 1) (*p* < 0.05).

A high body mass index, a younger age at menarche, a low PDG level during the early follicular phase, a small maximum follicle size, and a low PDG level during the periovulatory phase were all significantly associated with a higher risk of low mPDG (Outcome 2) (*p* < 0.05).

A small maximum follicle size and a high PDG level during the periovulatory phase were significantly associated with a three-fold higher risk of having a normal then low PDG level (Outcome 3) (*p* < 0.05).

A high PDG level during the early follicular phase and a high PDG level during the periovulatory phase were significantly associated with a three-fold lower risk of low then normal PDG (Outcome 4) (*p* < 0.05).

### Results of the multivariate analysis

#### Risks of short luteal phase (Outcome 1)

The higher risk of a short luteal phase in case of long preovulatory phase and high PDG level during the periovulatory phase persisted after adjustment for general, preovulatory, and periovulatory characteristics.

The higher risk of short luteal phase in case of small maximum follicle size persisted after adjustment for preovulatory characteristics but not after adjustment for the general or the periovulatory characteristics.

The risk of short luteal phase decreased significantly along with aging after adjustment for the general characteristics.

#### Risks of low mPDG (Outcome 2)

The lower risk of low mPDG in case of high PDG level during the periovulatory phase persisted after adjustment for the general, the preovulatory, and the periovulatory characteristics.

The lower risk of low mPDG in case of high age at menarche persisted after adjustment for preovulatory and periovulatory characteristics but not after adjustment for the general characteristics.

The lower risk of low mPDG in case of high early follicular phase PDG persisted after adjustment for the general and the preovulatory characteristics but not after adjustment for the periovulatory characteristics.

The higher risk of low mPDG in case of high body mass index persisted after adjustment for preovulatory and periovulatory characteristics but not after adjustment for the general characteristics.

The higher risk of low mPDG in case of small maximum follicle size persisted after adjustment for only the periovulatory characteristics.

#### Risks of normal then low luteal PDG level (Outcome 3)

The higher risk of normal then low PDG level in case of small maximum follicle size and high PDG level during the periovulatory phase persisted after adjustment for the general, the preovulatory, and the periovulatory characteristics.

After adjustment for the periovulatory characteristics, the risk of normal then low PDG level during the luteal phase decreased significantly in case of high PDG levels during the early follicular phase.

#### Risks of low then normal PDG level (Outcome 4)

The lower risk of low then normal PDG in case of high early follicular phase PDG persisted after adjustment for the general and the preovulatory characteristics but not after adjustment for the periovulatory characteristics.

The lower risk of low then normal PDG in case of high PDG level during the periovulatory phase persisted after adjustment for the general, the preovulatory, and the periovulatory characteristics.

After adjustment for the periovulatory characteristics, the risk of low then normal PDG during the luteal phase decreased significantly in women with high body mass index.

## Discussion

The first three outcomes (short luteal phase, low mPDG, and normal then low PDG level) were all more frequent in case of small vs. large maximum follicle size. The relationships were strong given that the frequency of each outcome was two to three times more frequent in case of small maximum follicle size. The relationships between a small maximum follicle size and a short luteal phase or a low mPDG did not persist when the regression is adjusted for women's general characteristics but the relationship between a small maximum follicle size and a normal then low PDG level remained whatever the variables used for adjustment. A high PDG level during the early follicular phase and a high periovulatory PDG level were protective against low mPDG and low then normal PDG level but a high periovulatory PDG level was followed by a significant increase in two outcomes: short luteal phase and normal then low PDG level. A low age at menarche and a high BMI were both predictors of low mPDG. We found no predictor of increased risk of low then normal PDG level; i.e., of slow luteinization. This outcome was less frequent in cases of high vs. low early preovulatory or periovulatory PDG levels. Finally, as expected, a long preovulatory phase was a predictor of a short luteal phase, with or without adjustment for other predictors.

These results support five general statements: (1) several factors known to be predictors of luteal phase deficiencies are also predictors of suboptimal luteal phase: some ovulation disorders that lead to luteal phase deficiencies could be exacerbations or deviations from an average cycle; (2) a short luteal phase, a low mPDG, and a normal then low PDG levels occur in case of small maximum follicle size: these may be signs of abnormal follicle development; (3) a low then normal PDG level appears to be a regulation process because the delay to reach a normal PDG level is longer in cycles with low vs. high PDG levels; thus, a low then normal PDG level is not a sign of abnormal luteal phase; (4) a high periovulatory PDG level (i.e., a premature luteinization) might be detrimental to the luteal phase that is then more frequently short and shows normal then low PDG levels; (5) in our population of normally fertile women, BMI was not found to be a predictor of luteal phase abnormalities but simply associated with lower mPDG levels.

Regarding the first statement, our results support the concept of a continuum from normal to abnormal cycle. Originally proposed by Brown (1), this concept was further supported by several studies (2–6). The causal processes of luteal phase deficiencies have not been fully explored to date. Some luteal phase deficiencies may be elements of the polycystic ovarian syndrome. Others might be variations of the normal physiological process. In addition, some authors have outlined the impact of environmental factors such as air pollution (10) or light pollution (11) that deserve specific attention due to their seemingly great impact on the physiology of reproduction (12). We have noticed that the relationships between a small maximum follicle size and a short luteal phase or a low mPDG are not maintained when the regression is adjusted for the general characteristics of the women; thus, small follicles and luteal deficiencies might be the consequences of an underlying characteristic. However, the relationship between a small maximum follicle size and a normal then low PDG level is maintained whatever the adjustment; thus, there might be a direct effect of follicle development on corpus luteum because a small corpus luteum stemming from a small follicle may have a short lifespan. These two propositions need to be confirmed by other studies.

Second, it has been asserted that luteal deficiencies originate from impaired follicular development (8, 13–15). The current analysis is in favor of this assertion. The maximum size of the follicle was shown only weakly correlated with the hormonal profile of the luteal phase (6). But, in the present analysis, using a binary variable to distinguish small from large follicles (any size >20 mm) led to observe a strong relationship between a small follicle size and three outcomes of suboptimal luteal phase (Outcomes 1–3). This has two potential implications: (i) use of these three outcomes for diagnostic purposes as proxies for abnormally small follicle size at ovulation when this information is not available from ultrasound examination; (ii) support the treatment of the ovulation process itself in case of luteal deficiency (8).

Third, in a previous publication (6), a long luteinization process was found to be frequently associated with a subsequent normal or high level of progesterone and a long luteal phase but there was no argument to support that low then normal progesterone is a sign of luteal deficiency. The present analysis of the same data but with other statistical methods provides the latter argument. In normally fertile women, a low then normal progesterone level seems to be a sign of long luteinization but not a sign of deficiency of the luteal phase.

Fourth, in our dataset, a high periovulatory PDG level was clearly correlated with a short luteal phase and a normal then low progesterone level and this relationship persisted after adjustments. These rather strange results deserve to be confirmed by other studies. Actually, the rarity of large studies with ultrasound confirmation of the day of ovulation as reference day might be the reason for which this result was not previously published.

Fifth, in our dataset, BMI was not found to be a predictor of luteal abnormality but was simply associated with low PDG levels. In previous studies, luteal phase deficiencies were frequent when a high BMI was part of a polycystic ovarian syndrome and ovulatory dysfunction was frequent when BMI was clearly out of the normal range (either too low or too high) (16). In our dataset most BMIs were in the normal range and BMI was found inversely correlated with PDG level at each phase of the cycle: early follicular, periovulatory, and mid-cycle. A general effect of increased aromatization of androgens to estrogens in adipocytes may indirectly influence the secretion of progesterone. A larger population with higher BMIs would clarify further the relationships between BMI and luteal phase characteristics.

Finally, a previous analysis of the same dataset (6) has reported that the length of the preovulatory phase did not predict the length of the early, the mid, or the late luteal phase. However, the use of binary variables to distinguish luteal phases shorter and longer than 12 days led to a positive relationship between a short luteal phase and a long preovulatory phase. Thus, this well-known relationship in infertility or polycystic ovarian syndrome is also observed in normally fertile women.

The present study confirmed that abnormal luteal phases are associated with short duration, low progesterone level, and small maximum follicle size, which seem to point to abnormalities in follicular development. However, the results do not confirm that a low then normal PDG level is a sign of a suboptimal luteal phase. This might be simply due to the need for more time to reach an appropriate level of progesterone secretion. The absolute value of the PDG level needs to be interpreted within the context of the entire cycle. It seems to be more valuable to compare pre- vs. post-ovulatory PDG levels rather than interpret a single PDG level at some time point of the cycle.

Our study provides this information, although, in the future, a confirmation with larger sample sizes would be helpful.

One limitation of this work is that we assumed we were studying normally fertile women, whereas this might not have been the case because some women were over 40 years old, and because some women in our sample had no previous live births. Nevertheless, <10% of the women were older than 40 years of age and <20% were nulliparous. Some cases of secondary infertility among women who are already mothers cannot be excluded, but this limitation is common to most studies in the same field.

Finally, we may conclude with some implications for medical diagnosis and treatment. First, the types proposed by Hilgers (8) to identify luteal phase defect might be more extensively used: it seems important to add to the well-known types (short luteal phase and low mid-luteal-phase progesterone levels) Hilgers' third type: an early drop in progesterone level. Moreover, we might consider more systematically two options in case of luteal deficiency: luteal phase support or treatment of follicle insufficiency. The main clinical implication of an abnormal luteal phase relates to infertility or to recurrent pregnancy loss. Future studies are required to determine ideal protocols for supporting the luteal phase; e.g., supplementation with progesterone or luteal phase HCG treatment (17). The data presented here may also assist in determining the clinical relevance of serum progesterone measurements at different time points of the cycle and how these progesterone levels could be interpreted.

## Author contributions

SA carried out the analyses. TB, RL, and PB participated to the discussion and reviewed the manuscript. JI revised the final drafts of the manuscript. RE designed the study, supervised the analyses, and drafted the manuscript.

### Conflict of interest statement

The authors declare that the research was conducted in the absence of any commercial or financial relationships that could be construed as a potential conflict of interest. The reviewer FB and the handling Editor declared their shared affiliation.

## Abbreviations

mPDG: mid-luteal phase PDG level
US-DO: ultrasound (determined) day of ovulation.
